# Placental Expression Patterns of Galectin-1, Galectin-2, Galectin-3 and Galectin-13 in Cases of Intrauterine Growth Restriction (IUGR)

**DOI:** 10.3390/ijms17040523

**Published:** 2016-04-07

**Authors:** Stefan Hutter, Julia Knabl, Ulrich Andergassen, Simone Hofmann, Christina Kuhn, Sven Mahner, Petra Arck, Udo Jeschke

**Affiliations:** 1 Department of Gynecology and Obstetrics, Ludwig Maximilians University of Munich, Maistraße 11, 80337 Munich, Germany; Stefan.hutter@med.uni-muenchen.de (S.Hu.); Julia.knabl@gmx.de (J.K.); Ulrich.andergassen@med.uni-muenchen.de (U.A.); simone.hofmann@med.uni-muenchen.de (S.Ho.); Christina.kuhn@med.uni-muenchen.de (C.K.); sven.mahner@med.uni-muenchen.de (S.M.); 2 Department of Gynecology and Obstetrics, University of Hamburg, Martinistr. 52, 20246 Hamburg, Germany; p.arck@uke.de

**Keywords:** galectins, placenta, trophoblast, decidua, IUGR

## Abstract

Galectins (gal) are members of the mammalian β-galactoside-binding proteins and recognize Galβ1-4GlcNAc and Galβ1-4GalNac (Thomsen-Friedenreich antigen (TF)) sequences of several cell surface oligosaccharides. In this study, gal-1, -2, -3 and -13 were investigated systematically in the trophoblast and decidua compartment of intrauterine growth restriction (IUGR) placentas and normal third trimester control placentas and stratified by fetal gender and gestational age. Within this study, 29 third trimester placentas after delivery were analyzed. Fetal gender was equally divided within both groups, and immunohistochemical staining was analyzed according to fetal gender and gestational age. Double immune-fluorescence with trophoblast-specific markers was used to identify galectin-expressing cells at the feto-maternal interface in the decidua. Gal-3 was significantly downregulated only in the extravillous trophoblast of IUGR placentas. In contrast, expressions of gal-2 and gal-13 were downregulated in both villous and extravillous trophoblast cells of IUGR placentas. In addition, gal-2 and gal-13 showed a highly correlated expression scheme in the placenta. There are significant gender-specific expression patterns for single prototype galectins with downregulation of gal-2 and gal-13 of male gender placentas in cases of IUGR. Gal-3 as the chimera type galectin shows only little gender-specific differences in expression, which disappear in IUGR cases.

## 1. Introduction

Galectins are broadly described in various tissues, and their attributed functions reach from immuno-modulation to regulation of metabolism [1,2]. They can be found in intra- and extra-cellular compartments and are involved in protein to protein interactions modulating cell growth, differentiation and apoptosis [3]. Their role in the uterus and in placenta, therefore in the feto-maternal interface, has been the interest of research over the last few years. Especially the aspects of reproductive medicine in terms of abortion, as well as pregnancy disorders, like preeclampsia (PE), in advancing pregnancy have drawn interest to galectins in general and to some galectins in particular concerning the proven or potential roles in pregnancy [4]. PE, still a major problem in perinatal medicine in both well- and poorly-developed countries, has been associated with various galectins, of which two by now appear to have more correlations than others: galectin-1 (gal-1) and galectin-13 (gal-13), also known as placental protein 13 (PP13). The latter is currently in clinical use for PE screening for high risk patients, as low levels can predict the incidence and extent of PE [5]. Gal-13 is detectable in maternal serum starting at seven weeks of gestation, and throughout pregnancy, its expression in syncytiotrophoblast is decreased [6]. This correlation has led authors to the idea of supplementing gal-13 in PE rats with the effect of attenuating the gestational disease, hence dealing with gal-13 as a potential cure [7]. Gal-1 expression has also been found to be decreased in decidua in cases of early PE (before 34 weeks of gestation), which matches the idea of gal-1 promoting vascular expansion during placentation via vascular endothelial growth factor (VEGF) [8]. Interestingly, gal-1 expression in cases of PE in the third trimester appears to be increased, hereby supporting the thesis of different etiologies for early and late onset PE [9]. It also takes an important role in feto-maternal tolerance, as it has been shown to reduce stress-induced abortion in a mouse model by inducing tolerogenic dendritic cells and CD4+CD25+IL-10+ regulatory T cells (Treg) when supplemented [10].

Galectin-2 (gal-2) has recently been described in placentas of PE patients and found to be decreased compared to control placentas, pointing at its potential role in the development of this gestational disease [11]. 

The condition of IUGR just like PE depends mostly on defective trophoblast invasion and impaired placental development in the first trimester [12]. Fetal growth restriction is based on inadequate oxygen and nutrient supply via the feto-maternal interface caused by insufficient vascularization, deficient glucose transport and placental amino acid transporters [13,14]. Chronic fetal hypoxia based on reduced blood flow in the uterine arteries and therefore within the placental vascular bed can be partially identified by Doppler flow indices [15]. Additionally, serum markers of different origins have been tested in regards to the predictability of fetal growth restriction. Amongst these, pregnancy-associated plasma protein-A (PAPP-A), activin A, placenta protein 13, also known as gal-13 and others have been suggested as promising predictive screening markers in this context [16]. However, by now, most of these first trimester markers, including gal-13, have not been shown to stand in significant correlation to the pregnancy outcome concerning growth restriction in an unselected maternal cohort [17]. Contrary to this finding, first trimester serum levels of PAPP-A are associated with placental morphometric changes in pregnancies complicated by IUGR and PE [16]. Other markers, like soluble fms-like tyrosine kinase-1 (sFlt-1) and placental growth factor (PlGF), are already in clinical use for PE diagnostics, and interestingly, the well-known sFlt-1/PlGF ratio was shown to be elevated in cases of IUGR to a similar extent as in cases of PE [18].

Summarizing today’s knowledge about IUGR pathology [19] and the role of several galectins in PE, which are closely linked to the development of IUGR, it appears conclusive to look for placental galectin expression in cases of IUGR as the first step in identifying possible correlations. Gender-specific expressions are of special interest, as this issue might elucidate further aspects of gender-specific differences in the outcome of IUGR cases. Furthermore, this could be the basis for fetal gender-specific treatments in this gestational pathology. 

## 2. Results

### 2.1. Gal-1 Expression Shows No Significant Changes in IUGR Placentas in Villous Trophoblasts

**Figure 1:**
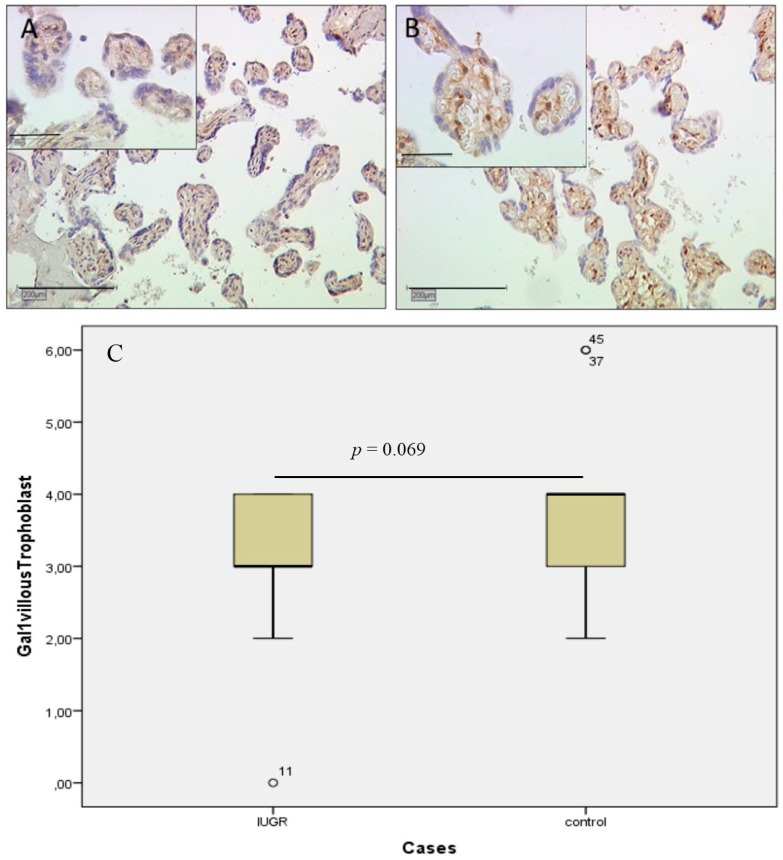
Staining of gal-1 in the villous trophoblast in cases of IUGR is shown in (**A**); for comparison with control placenta, see (**B**), showing only slight differences in staining intensity. The presentation of staining results is shown in (**C**) as a box-plot. The range between the 25th and 75th percentiles is represented by the boxes with a horizontal line at the median. The bars show the fifth and 95th percentiles. The circle indicates values more than 1.5 box lengths.

In villous trophoblast cells, gal-1 expression was identified by immunohistochemical evaluation. Staining scores of gal-1 evaluated with the International Remmele Score (IRS) in villous trophoblasts differ slightly in cases of IUGR (median IRS of three; Figure 1A) compared to control placentas (median IRS of four; Figure 1B). The presentation of staining results is shown in Figure 1C as a box-plot. There is no statistically-significant difference in staining results (*p* = 0.069). Gender-specific analysis has not shown any significant differences either.

### 2.2. In Villous and Extravillous Trophoblasts, Gal-2 Shows a Fetal Gender-Specific Expression in Control and IUGR Placentas

**Figure 2:**
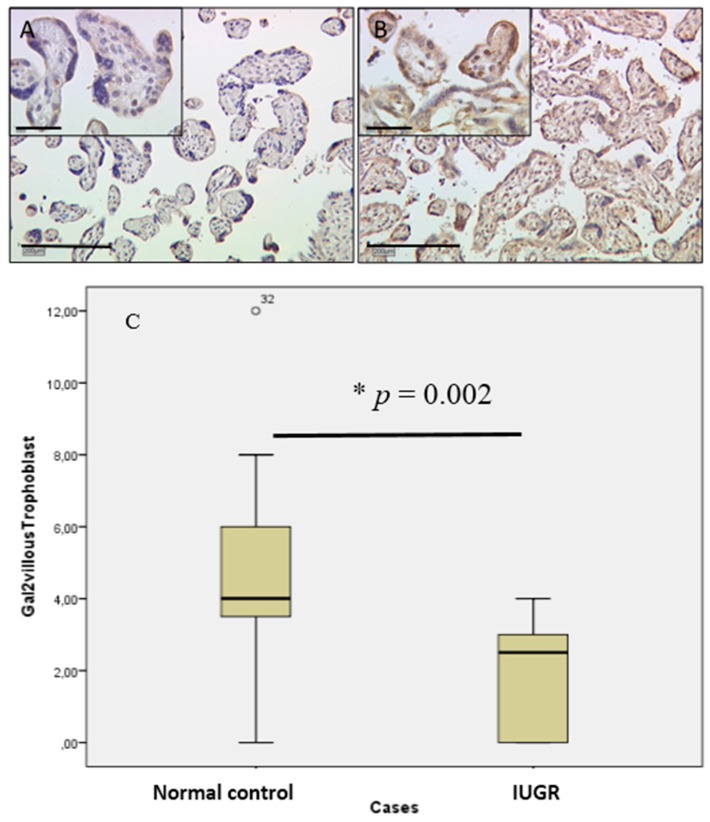
Gal-2 staining in cases of IUGR in villous trophoblasts (**A**) compared to control placentas (**B**) [20] appeared to be downregulated. The noted differences in staining intensity are statistically significant (* *p* = 0.002), as shown in the box-plot (**C**). The range between the 25th and 75th percentiles is represented by the boxes with a horizontal line at the median. The bars show the fifth and 95th percentiles. The circle indicates values more than 1.5 box lengths.

In villous and extravillous trophoblasts, gal-2 expression can be confirmed by the staining results. Using the IRS, upregulation of gal-2 expression in villous trophoblasts of male gender placentas compared to female gender is indicated. The scoring showed a two-fold increase in gal-2 staining in male cases (median IRS of six; Figure 2A) compared to female control placentas (median IRS of three; Figure 2B). The noted differences in staining intensity are statistically significant (*p* = 0.007), as shown in the box-plot (Figure 2C). In cases of IUGR, there was no change in expression in female gender placentas compared to controls; however, expression of gal-2 in male IUGR placentas showed a six-fold decrease (IRS of one) compared to male control placentas (IRS of six).

**Figure 3:**
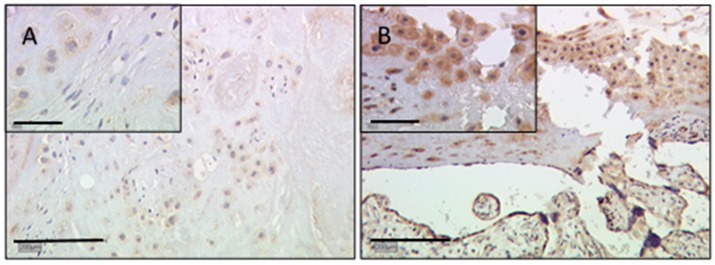

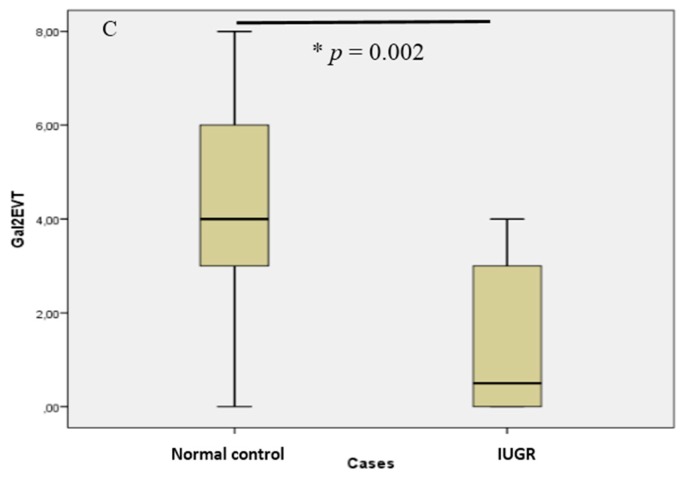
In extravillous trophoblasts, gal-2 expression appeared also to be decreased in IUGR placentas (**A**) compared to control placentas (**B**); Immunostaining revealed a statistically-significant four-fold decrease (* *p* = 0.002) in IUGR cases in comparison to control placentas, as shown in the box-plot presentation (**C**). For details of the box-plot interpretation, see the legend of Figure 2.

Gal-2 expression in extravillous trophoblasts appeared also to be increased in male control placentas compared to female control placentas (IRS of six *vs.* three, respectively). Immunostaining revealed a six-fold decrease in male IUGR cases (median IRS of one; Figure 3A) in comparison to male control placentas (median IRS of six; Figure 3B). Results were statistically significant (*p* = 0.004). In female gender placentas, there was no difference in expression between control and IUGR placentas. However, male IUGR placentas (IRS of one) displayed significantly decreased expression of gal-2 compared to female gender placentas (IRS of three). A summary of the scoring result is shown in box-plot presentations (Figure 3C).

### 2.3. In Extravillous Trophoblasts, Gal-3 Gender-Specific Differences in Expression Disappear in Cases of IUGR

**Figure 4:**
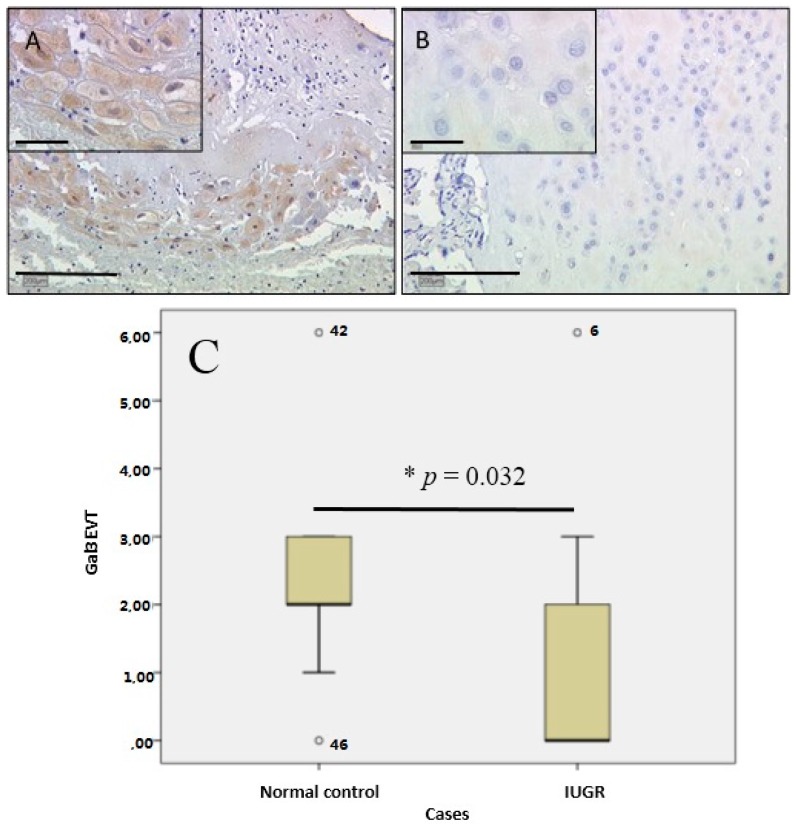
Gal-3 showed intermediate staining intensity in extravillous trophoblasts for control placentas (**A**); in cases of IUGR, extravillous trophoblast staining revealed a totally absent expression in 60% of the cases with a median IRS of zero (**B**); these results are statistically significant with * *p* = 0.032, shown in the box-plot presentation (**C**). For details of the box-plot interpretation, see the legend of Figure 2.

Staining of extravillous trophoblast for gal-3 showed intermediate staining intensity for control placentas in female (median IRS of two) and male gender placentas (median IRS of three) with a significant upregulation in male placentas (Figure 4A). However, in cases of IUGR, extravillous trophoblasts revealed a very weak expression in both gender-specific groups (median IRS of one and 0.5 for female and male, respectively; Figure 4B). These results are still statistically significant with *p* = 0.037, shown in box-plot presentation (Figure 4C).

### 2.4. Gal-13 Expression Is Strongly Decreased in Villous and Extravillous Trophoblasts in IUGR Complicated Pregnancies of Male Fetal Gender

**Figure 5:**
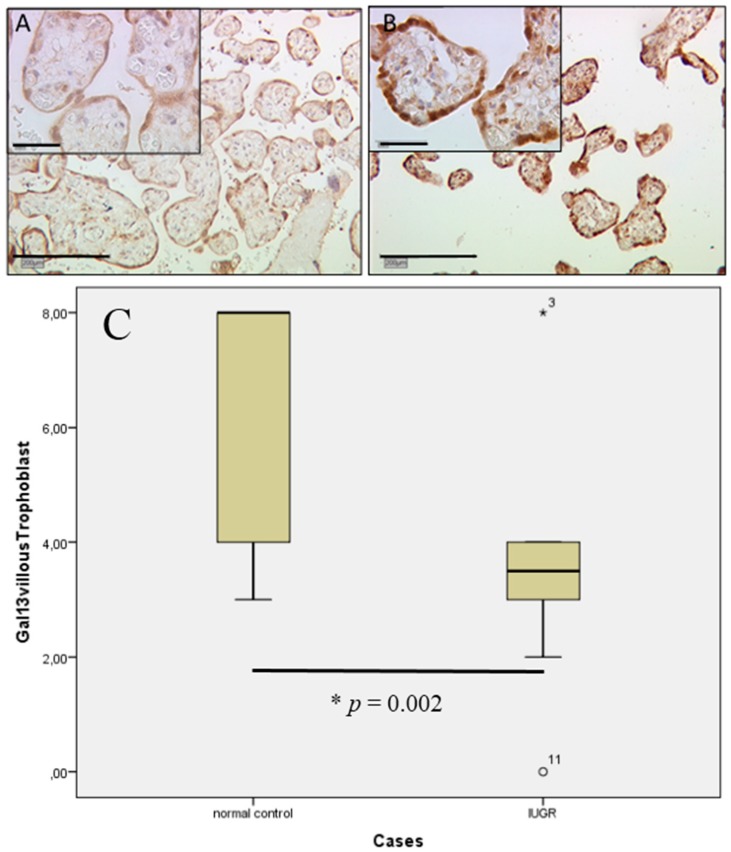
In villous trophoblast gal-13 expression is down regulated in cases of IUGR (**A**) compared to control placentas with high expression of gal-13 (**B**); The results in staining intensity are statistically significant (* *p* = 0.002) as shown in the Box-plot (**C**). For details of Box-plot interpretation see legend of Figure 2, and the asterisk indicates values more than 3.0 box lengths from the 75th percentile.

In villous and extravillous trophoblasts, strong gal-13 expression can be confirmed by staining results. Gal-13 expression in control placentas is upregulated in male gender villous trophoblasts, which is shown after applying the IRS (median IRS of eight) compared to female gender placentas (median IRS of four). In male fetal gender, cases of IUGR expression of gal-13 (median IRS o three; Figure 5A) appeared decreased compared to male control placentas with high expression of gal-13 (median IRS of eight; Figure 5B). The results in staining intensity for male gender placentas are statistically significant (*p* = 0.022), as shown in the box-plot (Figure 5C). Female gender placentas showed no significant difference between IUGR and control cases (median IRS of four for both; *p* = 0.054).

**Figure 6:**
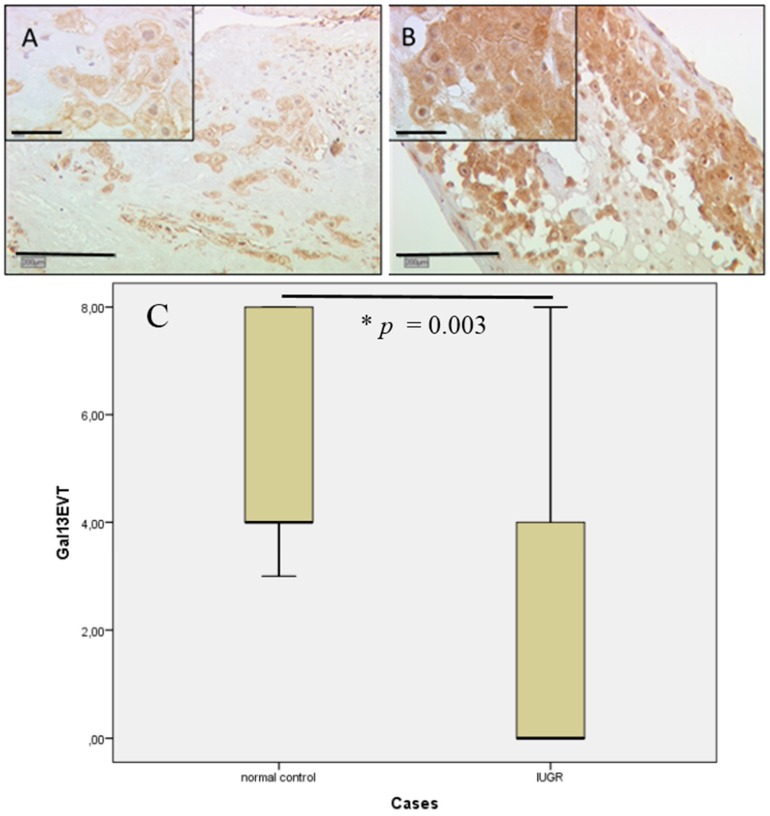
In IUGR placentas, gal-13 expression in extravillous trophoblasts was rarely detectable. IRS scoring revealed only weak to no expression (**A**); control placentas showed intermediate expression for gal-13 (**B**); again, all results were statistically significant (* *p* = 0.003). In summary the scoring result is presented in box-plot form (**C**). For details of the box-plot interpretation, see the legend of Figure 2.

Gal-13 expression in extravillous trophoblasts was only scarcely detectable in male IUGR placentas (median IRS of zero; Figure 6A), therefore showing a strong decrease in comparison to male control placentas (median IRS of eight). Female fetal gender placentas showed once more no significant difference between the control and IUGR groups (median IRS of four in both groups). In comparison, control placentas showed a strong expression for gal-13 male cases (median IRS of eight; Figure 6B) compared to female cases (median IRS of four). In summary, the scoring result is presented in box-plot form (Figure 6C).

### 2.5. Immunofluorescence Double Staining

**Figure 7:**
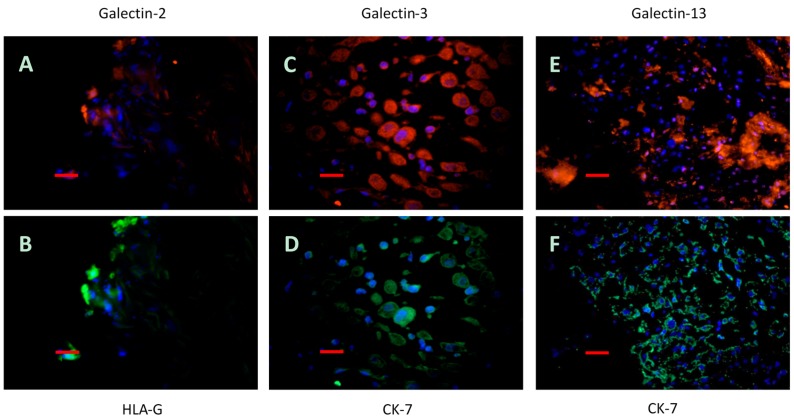
Gal-2 (**A**) (marked in red color) was found exclusively expressed by extravillous trophoblast cells marked with an HLA-G antibody (**B**); marked in green. A similar expression schema was found for gal-3 in the decidua. Gal-3 (**C**); also with red staining, showed a strong co-expression with CK-7 (**D**); shown in green. In addition, gal-3 is also expressed by cells negative for CK-7. Gal-13 staining in red (**E**) was found co-expressed by extravillous trophoblast cells marked with a CK-7 antibody in green (**F**). Scale bar, 500 µm in all cases.

For the characterization of galectin-expressing cells, immunofluorescence double labelling experiments were performed. HLA-G or CK-7 were used as markers for the extravillous trophoblasts. Galectin-2 (Figure 7A) was found exclusively expressed by extravillous trophoblast cells marked with an HLA-G antibody (Figure 7B). A similar expression schema was found for galectin-3 in the decidua. Galectin-3 (Figure 7C) showed a strong co-expression with CK-7 (Figure 7D). In addition, galectin-3 is also expressed by cells negative for CK-7. These cells can be regarded as decidual stromal cells. Finally, galectin 13 staining (Figure 7E) was found co-expressed by extravillous trophoblast cells marked with a CK-7 antibody (Figure 7F).

### 2.6. Correlation Analysis

To evaluate the co-expression of galectins in different parts of the placenta, we performed correlation analysis:

In the villous trophoblasts, we found no statistically-significant correlation of galectin-1 and galectin-2 (Spearman correlation coefficient *r* = 0.498; *p* = 0.059). However, a significant correlation was identified between galectin-2 and galectin-13 in villous trophoblast tissue (Spearman correlation coefficient *r* = 0.659; *p* = 0.008).

In the extravillous trophoblasts, we identified a significant correlation between galectin-1 and galectin-2 (Spearman correlation coefficient *r* = 0.708; *p* = 0.003) and between galectin-1 and galectin-13 (Spearman correlation coefficient *r* = 0.676; *p* = 0.006). In addition, galectin-2 showed a significant correlation with galectin-13 (Spearman correlation coefficient *r* = 0.620; *p* = 0.014). Galectin-3 showed no significant correlation to any other galectin in the healthy control placenta.

In IUGR placentas, a significant correlation was identified between galectin-2 and galectin-13 in villous trophoblast tissue (Spearman correlation coefficient *r* = 0.694; *p* = 0.006). 

In the extravillous trophoblast of IUGR placentas, we identified a significant correlation between galectin-2 and galectin-3 (Spearman correlation coefficient *r* = 0.658; *p* = 0.010). In addition, a very strong correlation was identified between galectin-2 and galectin-13 (Spearman correlation coefficient *r* = 0.902; *p* < 0.001).

### 2.7. Stratification for Gestational Age

**Figure 8:**
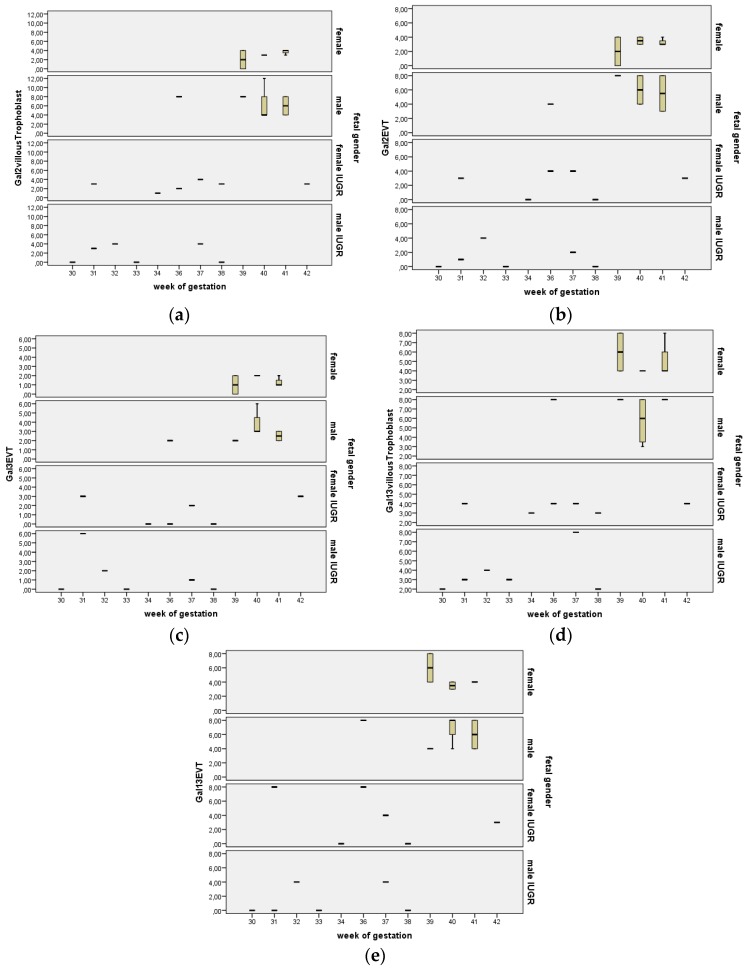
Stratification for gestational age does not reveal any dependency of galectin expression levels on progressing gestational age, as depicted for gal-2 in villous (**a**) and extravillous trophoblasts (**b**), for gal-3 in EVT (**c**) and gal-13 in both villous (**d**) and extravillous trophoblasts (**e**).

Stratification for gestational age is shown as a box plot diagram in Figure 8. The statistical analysis does not reveal any dependency of galectin expression levels on progressing gestational age as depicted for gal-2 in villous (Figure 8a) and extravillous trophoblasts (EVT, Figure 8b), for gal-3 in EVT (Figure 8c) and gal-13 in both villous (Figure 8d) and EVT (Figure 8e).

### 2.8. Production of Gal-2 mRNA Is Downregulated in Male IUGR Placentas

**Figure 9:**
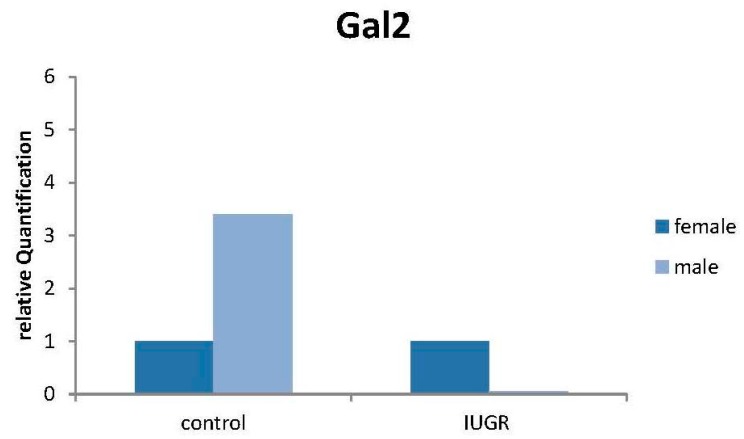
Male IUGR placentas showed a significantly lower production (3.5-fold downregulation) of gal-2 mRNA compared to the control placentas analyzed, with β-actin as the housekeeping gene.

Real-time RT-PCR results showed significant differences between male IUGR and control placentas’ gene production of gal-2 mRNA. Male IUGR placentas showed a significantly lower expression compared to control placentas both with β-actin (*p* = 0.020; Figure 9) as housekeeping genes. Quantification of gal-2 mRNA revealed a 3.5-fold downregulation of gal-2 mRNA in male IUGR compared to normal control placental tissue.

For gal-3, we identify a trend of a downregulation in female placentas without reaching significance. There was no downregulation on mRNA level observed for gal-13 expression.

## 3. Discussion

Overlooking the results concerning the prototype galectins described, there appears to be a strong fetal gender-specific expression pattern with the galectins 2 and 13, whereas galectin-1 does not show gender-specific changes in expression. The chimera type galectin-3, however, displayed only small, yet significant gender-specific differences in control placentas and none in IUGR placentas.

So far, gender-specific differences in placental tissue have been described in other pregnancy disorders, such as gestational diabetes for downregulation of estrogen receptor α in decidual vessels [21] and for tandem repeat galectins in cases of IUGR [22]. The experienced clinician is used to sexually dimorphic differences in pregnancy development, as male fetal sex is seen as an independent risk factor for adverse pregnancy outcome [23].

Some authors consider gender-specific intrauterine growth strategies to increase fetal vulnerability to under nutrition, as male fetuses tend to less placental growth [24]. Taking into account many other meaningful results of recent years, it is broadly recommended to account for sexual dimorphisms when dealing with fetal and placental reactions to maternal nutrition [25]. Nevertheless, it has to be stated that our results can only provide insight into placental expression at the time of delivery, and it appears necessary to take a closer look at the specific galectin expression patterns in the course of pregnancy. 

Expression of gal-2 and gal-13 both in villous and extravillous trophoblast cells was decreased in placentas of male fetal gender IUGR cases, and furthermore, both galectins showed similar expression patterns, as in all cases, expression in female gender placentas remained the same in control and IUGR. So far, gal-13 serum levels have been shown to be significantly decreased in first trimester pregnancies that later developed IUGR. This effect was also shown in pregnancies that were furthermore complicated by PE or preterm delivery [26]. As gal-13 is known to promote prostacyclin secretion in order to stimulate vascular remodelling of maternal spiral arteries in placental development, this finding is very conclusive [27]. Additionally, gal-13 is involved in trophoblast migration towards the placental bed via binding to beta and gamma-actin [28]. These effects concerning trophoblast development have been described in various ways, and decreased expression of gal-13 has also been shown in all placenta compartments of patients with gestational diabetes [29]. However, by now, gal-13 has not been implemented as a solitary or combined marker, neither for PE, nor for IUGR, lacking accuracy for screening purposes [17]. Overlooking recent studies concerning gal-13 levels in fetal growth restriction and PE, some potential reasons for missing accuracy and comparability could be found in the heterogeneous and multifactorial etiology of these disorders and missing normative levels to allow comparison in between heterogeneous populations [30]. A longitudinal study on gal-13 maternal serum levels once more confirmed low levels in small-for-gestational-age pregnancies, but did not reveal gender-specific differences. Though, these results have to be interpreted cautiously, as maternal weight has been shown as a strong confounding factor [31]. Concerning IUGR, gender-specific expression of this galectin might also be responsible for missing accuracy, and further research is needed to draw conclusions on maternal serum levels of specific galectins, as well. As our results are of a descriptive nature and mostly based on a semi-quantitative scoring system, it is difficult to draw functional conclusions; however, there are significant gender-specific differences that should be considered furthermore. 

As mentioned above, gal-2 expression was shown to be decreased in male IUGR placentas in all compartments when compared to controls. This finding matches partially the results concerning placental gal-2 expression in cases of PE, which in many cases is also based on impaired placentation [11]. However, in analogy to PE, it is still to be elucidated whether the decrease in gal-2 expression is causative for the development of IUGR or if it is a reaction to failed trophoblast invasion [32]. Even more interestingly, the question arises, whether there are gender-specific differences in cases of PE or not. The role of gal-2 as an inhibitor of arteriogenesis has been identified in a mouse model by showing its interference with the monocyte/macrophage population, leading to a low capacity of vascular remodelling [33]. Contrary to the findings of gal-13 and gal-2, the expression pattern of gal-1 does not differ significantly between IUGR villous trophoblasts and placental control tissue. Additionally there is no gender-specific dimorphism in gal-1 expression in villous trophoblasts. In early pregnancy, gal-1 is supposed to promote vascular expansion via vascular endothelial growth factor 2 (VEGF 2) at the decidua, and in this context, low levels of gal-1 have been found in cases of early onset PE [8]. In cases of late onset PE, however, gal-1 expression in extravillous trophoblasts and decidual cells appeared to be upregulated, leading to the hypothesis of compensatory elevated levels in the third trimester [9]. The decrease of gal-3 expression was only found significant in the extravillous trophoblasts of female gender control placentas compared to male gender placentas in our study. In IUGR placentas, extravillous trophoblasts did not show significant gender-specific differences with an overall very low expression of gal-3. In cord blood of small-for-gestational-age infants, expression of gal-3 is significantly higher compared to appropriate-for-gestational-age infants [34]. Gal-3 has been attributed a predominantly pro-inflammatory character in various studies [35]. Its role in terms of inflammation appears to be ambivalent, though [36]. Its ambivalence is obvious in apoptosis, where intracellular gal-3 shows anti-apoptotic effects, while extracellular gal-3 induces apoptosis of various cells [37]. Gal-3 as the only known chimera type galectin might be elevated in cord blood of IUGR infants due to inflammation, because of chronic hypoxia of the fetus. Additionally, gal-3 expression has been proven to be regulated by hypoxia-inducible factor 1 (HIF-1) in several studies [38,39].

In general, the placental expression of galectins appears to be downregulated in cases of IUGR. Gal-2 and gal-13 seem to be highly correlated in their placental expression in all placental compartments. The expression scheme of gal-3, the only chimera type galectin, seems to be independent of the prototype galectins 1, 2 and 13. 

## 4. Materials and Methods

### 4.1. Placental Tissues

Placental tissues were obtained from 29 women giving birth in the Department of Obstetrics and Gynaecology of the LMU Munich. A total of 14 placentas with IUGR (mean date of delivery: 33.0 ± 3.0 weeks of gestation) and 15 normal placentas (mean date of delivery: 38.2 ± 3.9 weeks of gestation) were used for the study (see Table 1 for clinical outcome). Placental samples were stratified by gestational age and by fetal gender to allow the detection of gender-specific differences in galectin expression. For gender-specific analysis, 7 normal control placentas with female and 8 with male gender were used. For IUGR placentas, there were 7 placentas of male or female gender, respectively.

Fetal IUGR was defined as an estimated intrauterine weight below the 5th centile, and asymmetric fetal growth restriction after gestational age had been confirmed by an early pregnancy ultrasound. IUGR needed to be confirmed after delivery; otherwise, the patient was excluded from the study. 

The following exclusion criteria were applied for maternal conditions: chorioamnionitis, chronic hypertension, chronic renal disease, cardiac disease, connective-tissue disease, pre-existing diabetes mellitus or gestational diabetes mellitus. The study was approved by the local ethics committee of the LMU Munich, Germany (No. 158/00). Informed consent was obtained from all patients in written form.

Tissue samples were cut from the central part of the placenta directly after delivery. Visually ischemic and necrotic areas were excluded from sampling in favor of cotyledon structures showing sufficient blood supply. Samples, measuring 2 cm^3^, contained decidua, extravillous and villous trophoblasts and amniotic epithelial cells. After obtaining two neighboring samples, the tissue was frozen at −80 °C and stored, and one block was fixed in 4% neutral buffered formalin, dehydrated and consecutively embedded in paraffin. 

After cutting the specimens in 2–3-µm slices, they were mounted on SuperFrost/Plus microscope slides (Menzel, Walldorf, Germany) followed by dewaxing and rehydrating in a descending series of alcohol before using them for immunohistochemical antibody detection. 

### 4.2. Immunohistochemical Staining for Paraffin Sections

For immunohistochemical staining, specimens were treated in analogy to former protocols as used with tandem repeat galectins [22]. Tissue sections were then incubated with the primary antibody (Table 2) for 16 h at 4 °C in a humid chamber. After washing the sections 2 times with PBS before applying, post-block reagent (Zytomed Systems, ZytoChem Plus HRP Polymer System, Berlin, Germany, mouse/rat/rabbit) was applied. After 20 min of incubation at room temperature in a humid chamber, the sections were again rinsed 2 times with PBS, and then, the HRP-Polymer reagent (Zytomed Systems, ZytoChem Plus HRP Polymer System, mouse/rabbit) was applied for 30 min, followed by peroxidase staining reaction. This visualization reaction was performed using the chromogen 3,3’-diaminoenzidine (DAB; Dako, Glostrup, Denmark).

Staining of all specimens was accomplished in one session, so as to avoid inter-assay variability.

### 4.3. Evaluation of Staining

The specimens were evaluated using the semiquantitative International Remmele Score (IRS) [40]. The IRS is calculated by multiplying the optical staining intensity (graded as 0 = no staining, 1 = weak staining, 2 = moderate staining, 3 = strong staining) and the percentage of positively-stained cells (0 = no staining, 1 = <10% of cells, 2 = 11%–50% of cells, 3 = 51%–80%, 4 = >80% of cells stained).

### 4.4. Immunofluorescence Double Staining

Immunofluorescence double staining was prepared according to the protocol published earlier concerning tandem repeat galectins [22]. Galectin primary antibodies were applied either with HLA-G antibody or CK-7 antibodies using dilutions shown in Table 2. After incubation, the secondary antibodies Cy3-labeled goat anti-mouse IgG (1:100 in background reducing antibody diluent (Dako)) and Cy2-labeled goat anti-rabbit IgG (1:500 in background reducing antibody diluent (Dako)) were applied (both antibodies form Dianova, Hamburg, Germany). Alternatively, for gal-3, Cy3-labeled goat anti-rabbit IgG (1:100 in background reducing antibody diluent (Dako)) and Cy2-labeled goat anti-mouse IgG (1:500 in background reducing antibody diluent (Dako)) were used. Finally, the sections were embedded in mounting medium for fluorescence containing 4′,6-diamino-2-phenylindile (DAPI) to stain the nuclei.

### 4.5. Control Slides for Galectin Staining

**Figure 10:**
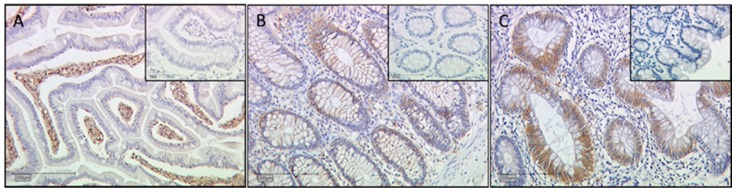
Control tissue for evaluation of gal-1 staining in duodenum (**A**); As a positive control for gal-2 and gal-3 expression, colon tissue was stained ((**B**,**C**), respectively). Appropriate negative controls are shown as inserts of each figure.

The positive control tissue for evaluation of galectin-1 staining is duodenum (Figure 10A) [41]. Galectin-2 expression was shown recently in human colon tissue [42]. As a positive control for Galectin-2 expression, colon tissue was stained (Figure 10B). Colon tissue was also used as a positive control for galectin-3 expression (Figure 10C) [43]. Galectin-13 expression was only described in placental tissue [29]. Negative controls are shown as inserts in each figure. Negative controls were stained according to positive controls with the exception that the primary antibody was replaced by species-specific isotype control IgGs.

### 4.6. Isolation of RNA for qPCR

Total RNA was obtained using the NucleoSpin^®^RNAII Kit (Macherey-Nagel; Düren, Germany Nr.740955.50) according to the manufacturer’s protocol. First, cell-lysis was induced with RA1 and β-mercaptoethanol (3 µL), and then lysate was filtered with the NucleoSpin-filter (Macherey-Nagel; Düren, Germany) followed by RNA binding performance and application of membrane desalting buffer. Finally, DNA was digested by adding rDNAse and reaction buffer for rDNA (1:10).

### 4.7. cDNA Synthesizing/Reverse Transcription

cDNA synthesizing was done by reverse transcription with the TaqMan-EZ RT-PCR Kit (PE, Applied Biosystems, Foster City, CA, USA). For the implementation of quantitative real-time PCR reactions (qPCR), 10 µL 2 x Reverse Transcription-Mastermix and 10 µL mRNA were added. The reaction was performed with the Eppendorf Mastercycler gradient (Eppendorf, Hamburg, Germany) for 10 min at 25 °C, 120 min at 37 °C, 5 s at 85 °C and 4 °C on hold.

### 4.8. qPCR

A total volume of 20 µL containing 1 µL TaqMan^®^ Gene Expression Assay 20 x (Table 3; Applied Biosystems), 10 µL TaqMan^®^ Fast Universal PCR Master Mix 2 x (Applied Biosystems), 1 µL cDNA template and 8 µL H_2_O (DEPC-treated DI water, Sigma, St. Louis, MO, USA) was applied per probe on an Optical Fast 96-well plate (Applied Biosystems) and covered by optical caps (Applied Biosystems). Using Taqman 7500 Fast (Applied Biosystems), PCR assays were performed. At 95 °C for 20 s on hold, enzymes were activated followed by 40 cycles of qPCR denaturing at 95 °C for 3 s and annealing at 60 °C for 30 s. The Relative Quantification (RQ) = 2^−∆∆*C*t^ method, which is defined as the first fluorescent signal reaching statistical significance, was applied for the results. ∆*C*_t_ values were calculated by normalizing to β-actin, which was used as an endogenous control.

### 4.9. Statistics

The SPSS/PC software package, Version 20 (SPSS GmbH, Munich, Germany), was used for data collection and processing, as well as analysis of statistical data. Correlation analysis was performed with the non-parametric Spearman’s rank correlation coefficient, which analyzes the statistical dependence between two monotonic, non-linear variables. Values with *p* < 0.05 were considered statistically significant. The Mann–Whitney *U*-test was used for the evaluation of two independent groups. These tests are the one-way analysis of variance and analyze two parameters that are independent from each other.

## 5. Conclusions

Our results concerning gal-13/PP13 expression match with former studies on its maternal serum level. Interestingly, IUGR infants showed elevated gal-3 concentration in cord blood, but decreased levels in maternal serum of multiparas. As there are different correlations between placental galectin expression and serum levels, which are still to be elucidated, many results concerning these aspects have to be treated cautiously. Obviously, there are explicit gender-specific expression patterns for single prototype galectins with a significant downregulation of gal-2 and gal-13 of male gender placentas in cases of IUGR. Gal-3 is a chimera type galectin and shows only little gender-specific differences in expression, which disappear in IUGR cases. Gal-1 does not show any sex-specific differences in control and IUGR placentas. It remains unclear which part sex-specific galectin expression takes in IUGR cases; however, better understanding of the underlying reasons and etiologies might lead to fetal sex-specific perinatal and prenatal management. Further studies, including animal models, are needed to fully elucidate the correlation and causal relationship between maternal serum levels, cord blood levels and placental expression of these galectins with special respect to gender-specific differences to fully understand their function in gestational pathologies.

## Figures and Tables

**Table 1:** Clinical details on the patients and newborns in the IUGR and normal control group.

Parameter	IUGR		Control	
	Male	Female	Male	Female
Duration of gestation at delivery (weeks)	33 (SD 3.27)	37 (SD 4.30)	40 (SD 1.6)	40 (SD 0.9)
neonatal birth weight (g)	1400 (SD 496.65)	1950 (SD 625.7)	3157.5 (SD 480.3)	3440 (SD 249.19)
pH umbilical artery	7.32 (SD 0.04)	7.36 (SD 0.08)	7.3 (SD 0.09)	7.39 (SD 0.08)
Apgar score at 5 min	9 (SD 1.52)	9.5 (SD 2.34)	10 (SD 0)	10 (SD 0)
Apgar score at 10 min	10 (SD 1.73)	10 (SD 1.6)	10 (SD 0)	10 (SD 0)
maternal age (years)	32 (SD 5.43)	31 (SD 3.45)	30,5 (SD 6.4)	33 (SD 5.20)
ethnicity of parents	Caucasian (100%)	Caucasian (100%)	Caucasian (100%)	Caucasian (100%)

SD: standard deviation, g: grams.

**Table 2:** Antibodies used in the study.

Antigen	Antibody	Isotype	Dilution	Source
Gal-1	201002	Rat IgG2b	1:50	R&D
Gal-2	NBP1-89690	Rabbit IgG	1:100	Novus Biologicals
Gal-3	9C4	Mouse IgG1	1:500	Novocastra
Gal-13	NBP1-91922	Rabbit IgG	1:50	Novus Biologicals
HLA-G	MEM-6/9	Mouse IgG1	1:50	Dako
CK7	NCL-L-CK7-OVTL	Mouse IgG1	1:30	Novocastra
CK7	Sc-25721	Rabbit IgG	1:100	Santa Cruz

**Table 3:** Primers and probes used for semi-quantitative mRNA detection.

Target	Applied Biosystems Number
LGALS2	Hs00197810_m1
LGALS3	Hs00173587_m1
LGALS13	Hs00747811_m1
ACTB	Hs99999903_m1

## Abbreviations

MDPI	Multidisciplinary Digital Publishing Institute
DOAJ	Directory of Open Access Journals
TLA	three letter acronym
LD	linear dichroism
